# Neonatal Nav1.5 Protein Expression in Human Colorectal Cancer: Immunohistochemical Characterization and Clinical Evaluation

**DOI:** 10.3390/cancers13153832

**Published:** 2021-07-30

**Authors:** Elena Lastraioli, Scott P. Fraser, R. Mine Guzel, Jessica Iorio, Lapo Bencini, Emanuela Scarpi, Luca Messerini, Vincenzo Villanacci, Giulia Cerino, Niccolo’ Ghezzi, Giuseppe Perrone, Mustafa B. A. Djamgoz, Annarosa Arcangeli

**Affiliations:** 1 Department of Experimental and Clinical Medicine, University of Florence, viale GB Morgagni 50, 50134 Florence, Italy; elena.lastraioli@unifi.it (E.L.); jessica.iorio@unifi.it (J.I.); luca.messerini@unifi.it (L.M.); Annarosa.arcangeli@unifi.it (A.A.); 2 Department of Life Sciences, South Kensington Campus, Imperial College London, London SW7 2AZ, UK; spfraser1965@gmail.com (S.P.F.); mguzel@biruni.com.tr (R.M.G.); 3 Department of Oncology, Division of Oncologic Surgery and Robotics, Azienda Ospedaliero-Universitaria Careggi, Largo Brambilla 3, 50134 Florence, Italy; bencinil@aou-careggi.toscana.it (L.B.); giulia.cerino@unifi.it (G.C.); niccolo.ghezzi@unifi.it (N.G.); 4 Unit of Biostatistics and Clinical Trials, IRCCS Istituto Romagnolo per lo Studio dei Tumori (IRST) “Dino Amadori”, Via P Maroncelli 40, 47014 Meldola, Italy; emanuela.scarpi@irst.emr.it; 5 Institute of Pathology, ASST Spedali Civili di Brescia, Piazzale Spedali Civili 1, 25123 Brescia, Italy; villanac@alice.it; 6 Pathology Unit, Campus Bio-Medico University, via A del Portillo 200, 00128 Rome, Italy; G.Perrone@unicampus.it; 7 Cyprus International University, Biotechnology Research Centre, Haspolat, Mersin 10, Cyprus

**Keywords:** colorectal cancer, metastasis, voltage-gated sodium channel, neonatal Nav1.5, immunohistochemistry

## Abstract

**Simple Summary:**

The voltage-gated sodium channel is a type of protein normally expressed in the ‘excitable’ tissues (nerves and muscles) of the body. Epithelial tissues (gut, lungs etc.), which are normally devoid of such a channel, express it at high levels upon becoming cancerous. This occurs also in colorectal cancer cells where the channel subtype is the embryonic (‘neonatal’) variant, nNav1.5. In colorectal cancer cells, as in other solid cancer cells, channel activity promotes invasiveness. However, there is little information on the status of nNav1.5 in human colorectal tissues and how this might relate to patient outcome. Here, we show (i) that nNav1.5 expression is much higher in cancer tissues compared with normal; (ii) that nNav1.5 co-occurs with several other biomarkers of pathological importance; and (iii) that disease-free survival of colorectal patients is inversely correlated with channel expression. In conclusion, nNav1.5 has combined diagnostic and therapeutic potential in clinical management of colorectal cancer.

**Abstract:**

Voltage-gated Na^+^ channels (VGSCs) are expressed widely in human carcinomas and play a significant role in promoting cellular invasiveness and metastasis. However, human tissue-based studies and clinical characterization are lacking. In several carcinomas, including colorectal cancer (CRCa), the predominant VGSC is the neonatal splice variant of Nav1.5 (nNav1.5). The present study was designed to determine the expression patterns and clinical relevance of nNav1.5 protein in human CRCa tissues from patients with available clinicopathological history. The immunohistochemistry was made possible by the use of a polyclonal antibody (NESOpAb) specific for nNav1.5. The analysis showed that, compared with normal mucosa, nNav1.5 expression occurred in CRCa samples (i) at levels that were significantly higher and (ii) with a pattern that was more delineated (i.e., apical/basal or mixed). A surprisingly high level of nNav1.5 protein expression also occurred in adenomas, but this was mainly intracellular and diffuse. nNav1.5 showed a statistically significant association with TNM stage, highest expression being associated with TNM IV and metastatic status. Interestingly, nNav1.5 expression co-occurred with other biomarkers associated with metastasis, including hERG1, KCa3.1, VEGF-A, Glut1, and EGFR. Finally, univariate analysis showed that nNav1.5 expression had an impact on progression-free survival. We conclude (i) that nNav1.5 could represent a novel clinical biomarker (‘companion diagnostic’) useful to better stratify CRCa patients and (ii) that since nNav1.5 expression is functional, it could form the basis of anti-metastatic therapies including in combination with standard treatments.

## 1. Introduction

Colorectal cancer (CRCa) represents a major health issue worldwide [1]. According to Globocan estimates for 2020, CRCa currently accounts for 10% of cancer incidence and for 9.4% of cancer-related deaths in both sexes worldwide [1]. Most CRCa cases are classified as adenocarcinomas, arising from a multistep process known as the “adenoma-carcinoma sequence” [2]. Adenomas can occur in the colonic epithelium and up to 95% of adenocarcinomas may develop from those adenomas bearing potentially malignant molecular characteristics and high-grade dysplasia [3]. This was supported by a recent study comparing molecular features (e.g., genomic, transcriptomic, and methylation profiles) of adenomas from CRCa patients with those from non-oncological cases [4]. According to the “Tumour-Node-Metastasis” (TNM) classification scheme, four CRCa stages can be identified [5]. TNM I, II and III are characterized by local disease whilst TNM IV patients have distant (mainly liver and/or lung) metastases [5]. TNM I tumours can be cured by surgery alone and TNM III patients are always treated with adjuvant chemotherapy after surgery, according to standard guidelines [6]. In contrast, TNM II patients are usually not given adjuvant chemotherapy and some patients may relapse and might therefore benefit from additional treatment [7]. TNM IV patients are generally treated by systemic approaches, including standard chemotherapy, targeted therapy, or some combination of these, although the surgery could represent a curative option for some. Although the current landscape of CRCa treatments can involve several different targeted therapy approaches, the treatment for advanced and metastatic disease is still complex and frequently of limited effectiveness [8]. In order to optimise treatment(s), several biomarkers have been proposed [8]. According to National Comprehensive Cancer Network (NCCN) guidelines published in 2017, therapeutic choices are highly dependent on tumour localisation together with molecular features, such as *RAS*, *EGFR,* and *BRAF* mutations; microsatellite instability, CpG island methylation status; expression of *SCNA,* and TS, P21, and PTEN proteins [9]. Of these, mainly *KRAS* and *EGFR* mutations are used clinically but relate only to some 40 and 3% of stage IV cases, respectively [10]. Consequently, there is significant unmet need related to the discovery of novel functional biomarkers of CRCa that can detect the risk of metastasis and enable improved therapies. In both regards, mounting evidence suggests that ion channels could serve as useful biomarkers of CRCa [11].

In particular, it has been demonstrated that several human cancers express functional voltage-gated Na^+^ channels (VGSCs) and that VGSC activity promotes cellular invasiveness and metastasis [12,13,14,15,16,17,18]. In CRCa, it is the VGSC subtype Nav1.5 (gene: *SCN5A*) that is dominant [14]. *SCN5A* is subject to developmentally regulated alternative splicing of exon 6, giving rise to two different isoforms of Nav1.5, namely nNav1.5 and aNav1.5, present mainly in ‘neonatal’ and ‘adult’ tissues, respectively [13,19]. These two isoforms are characterised by changes of several amino acids in the S3-S4 region of domain I of the protein [13]. This enabled the production of a polyclonal antibody (NESOpAb) with high selectivity to nNav1.5 [19]. In human CRCa cells, it was predominantly nNav1.5 that was found to be functionally expressed and to drive invasiveness [20]. In contrast to cellular studies, the status of nNav1.5 specifically in clinical tissues of CRCa and the potential relevance of the channel occurrence to the pathophysiology of CRCa are not known. Using the specific NESOpAb antibody, the main aims of the present study were (i) to evaluate the expression of nNav1.5 protein in human CRCa tissues; (ii) to relate this to their pathological features as well as to some other interesting biomarkers; and (iii) to evaluate the expression in relation to patient history and outcome.

## 2. Results

**Figure 1:**
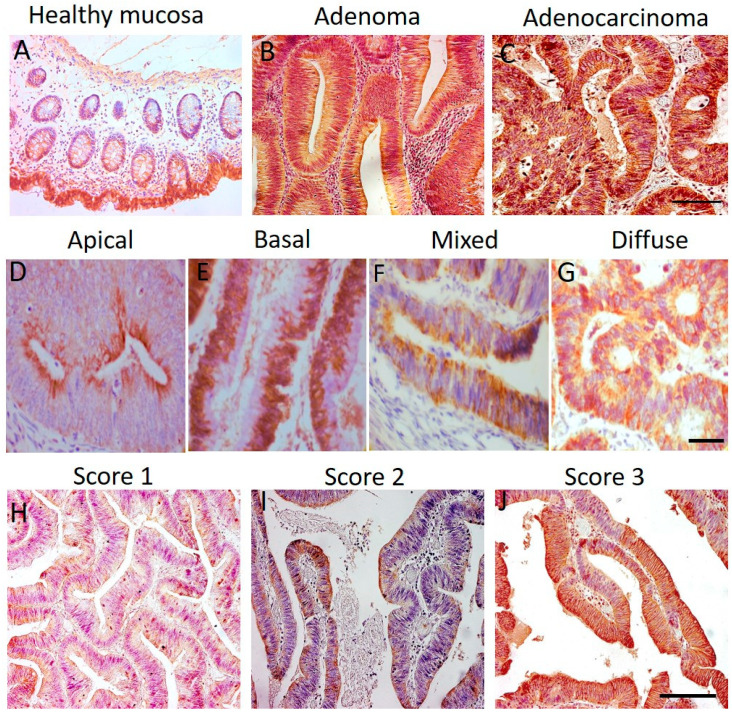
Expression of nNav1.5 protein in colorectal tissues. Immunohistochemistry was carried out using the polyclonal antibody, NESOpAb as described in Materials and Methods. In each picture, nuclei are counterstained in blue by haematoxylin, since nNav1.5 is not present in this cell compartment. (**A**) healthy mucosa, (**B**) adenoma, (**C**) adenocarcinoma. Scale bar: 100 µm. (**D**–**G**) Representative micrographs of adenocarcinomas showing different staining patterns: apical (**D**), basal (**E**), mixed (**F**) and diffuse/all-over (**G**). Scale bar: 50 µm. (**H**–**J**) Adenocarcinomas with different scores (1 to 3). Scale bar: 100 µm.

We analysed samples of 63 normal mucosa, 40 adenoma, and 182 adenocarcinomas encompassing all TNM stages. All the patients had undergone surgery for primary CRCa at the three different clinical institutions involved in the study. Representative examples of each type of tissue stained for nNav1.5 protein expression are shown in Figure 1A–C.

### 2.1. nNav1.5 Protein Expression

The following parameters representing different features of the immunostaining were quantified (details in Materials and Methods):

- Extent (E), defined as the percentage of stained cells in 20 randomly chosen fields of view;
- Staining Intensity (SI), scored as 0, 1 or 2 for no, weak and strong staining, respectively;
- Total Staining (TS), defined a SI × E; and
- Delineation Factor (DF), defined as [N_D_/20] × 100, where N_D_ is the number of fields of view (amongst the 20) showing overt ‘discrete’ staining. Accordingly, samples with diffuse, ‘all-over’ cytoplasmic staining would have a DF value near zero whilst those with discernible apical and/or basal membrane staining would have a higher DF value, maximum 100%.

As evident from Figure 1A, there was only very weak expression of nNav1.5 in healthy mucosa (TS = 0) and in only 20.6% (13/63) of cases. The pattern of the staining, when observed, was diffuse (median DF = 0%) (Table 1).

In adenocarcinomas, the nNav1.5 protein expression occurred in 71.4% (130/182) of cases. The immunostaining was (i) strong with a median value of TS = 60 a.u. (Figure 1C) and frequently ‘discrete’ with a median DF = 20% (Table 1A). Representative pictures of adenocarcinomas with differently patterned nNav1.5 immunostaining are shown in Figure 1D–G. Typical cases of scores 1, 2, and 3 are illustrated in Figure 1H–J.

In addition, for a subset of carcinomas (48 samples), matching mucosa tissues were available, and these were compared separately (Table 1B). The total staining as well as the delineation factor were again significantly higher for the adenocarcinomas vs. the corresponding mucosa: median TS = 25 vs. 0 a.u. and median D = 10 vs. 0%, respectively (Table 1B).

In the adenoma cohort, the immunostaining of nNav1.5 was surprisingly extensive. Thus, 87.5% (35/40) of samples displayed nNav1.5 protein expression (Figure 1B) and this was strong (TS = 160 a.u.; Table 1A). Importantly, however, the staining pattern was diffuse (DF = 0%; Table 1A). Dividing the adenomas into two groups (low- and high-grade dysplasia) and comparing their staining parameters did not reveal any difference (Table S1).

Overall, we concluded (i) that nNav1.5 immunostaining was more intense, widespread, and discrete in adenocarcinomas compared with healthy mucosa and (ii) that adenomas also expressed nNav1.5 protein but this was diffuse (Table 1).

### 2.2. Immunostaining for Other CRCa Biomarkers and Comparison with nNav1.5

The adenocarcinoma cohort was also analysed in relation to several other previously suggested CRCa biomarkers: hERG1 and KCa3.1 (potassium channels); carbonic anhydrase IX (CA IX); vascular endothelial growth factor-A (VEGF-A); glucose transporter 1 (Glut1); Ki67; P53; BCL2 and epidermal growth factor receptor (EGFR). nNav1.5 expression was positively associated significantly with the following: hERG1 (*p* = 0.046), KCa3.1 (*p* < 0.001), VEGF-A (*p* = 0.032), Glut1 (*p* = 0.041), and EGFR (*p* = 0.050) (Table S2).

### 2.3. Clinicopathological Considerations

Possible associations between nNav1.5 protein expression (including staining patterns) and patients’ clinical data for the adenocarcinoma cohort are shown in Table 2 and Table S3. Of the 182 patients, 94 (51.6%) were females and 88 (48.4%) were males. Median age was 70 years (range 37–91, IQR 61-78). Regarding location, 81 (44.5%) of the tumours were in the right colon, 11 (6.1%) in the transverse area, 51 (28.0%) in the left colon and 39 (21.4%) in the rectum. There was a statistically significant association between the level of nNav1.5 expression (“score”) and TNM stage with the highest score (score 3) occurring in stage IV (*p* = 0.005). Indeed, all score-3 samples belonged to the TNM IV class. Moreover, there was a statistically significant correlation between nNav1.5 expression and clinically detected metastases (*p* < 0.001; Table 2). These data are consistent with the nNav1.5 being a promoter of metastasis.

The possible association of the different staining patterns with clinicopathological features was also questioned (Table S3). A statistically significant association was observed only for lesions of the transverse colon where nNav1.5 expression was noticeably delineated (DF = 18%), partially localised to apical and/or basal membranes of the neoplastic cells (*p* = 0.027). 

### 2.4. Association of nNav1.5 Expression with Survival

Median follow-up was 24 months (range 1–120). Overall, 32 patients died during the period of the investigation. In univariate analyses, several parameters (age, localisation, presence of metastases, nNav1.5 and Glut-1 expression) showed statistically significant associations with progression free survival, PFS (Table 3). Among these, the presence of metastases showed the strongest correlation (*p* = 0.0008, HR: 4.06, 95% CI: 1.79–9.34). Notably, nNav1.5 expression also had a significant impact on PFS (*p* = 0.031, HR: 4.64, 95% CI: 0.93–23.03). For overall survival (OS), only Glut-1 appeared to be significantly associated (*p* = 0.042, HR: 0.39, 95% CI: 0.16–0.97) (Table 3).

A multivariate analysis was also performed and a model was built containing the variables that showed statistical significance in the univariate analysis. In the multivariate analysis, only Glut1 persisted in being significantly correlated with OS (*p* = 0.016; HR: 0.30, 95% CI: 0.11–0.80).

**Figure 2:**
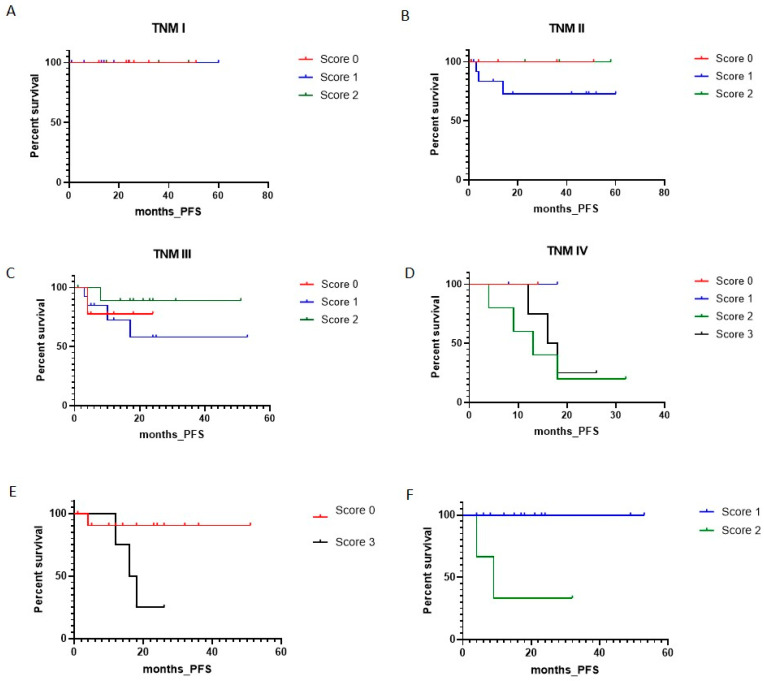
Kaplan-Meier plots of PFS comparing different nNav1.5 scores. (**A**–**D**), all scores in the four TNM stages. (**E**), score 0 vs. score 3 in all TNM stages. (**F**) score 1 vs. score 2 in left colon. Score 0: red curves; Score 1: blue curves; Score 2: green curves; Score 3: black curves.

We then evaluated whether the association of nNav1.5 with PFS could be more relevant in certain conditions. For this, we took together the data showing statistically significant associations between nNav1.5 expression and clinical features (Table 2) and the results of the univariate analysis. Thus, different analyses were carried out evaluating the nNav1.5 score in the context of TNM and tumour localisation (Figure 2).

There was no correlation between the nNav1.5 score and PFS for the TNM stages tested individually (Figure 2A–D). Nevertheless, higher scores (2 and 3) were associated with poorer prognosis in stage IV (Figure 2D). A significant impact of nNav1.5 expression on PFS emerged when comparing scores 0 and 3 for all TNM stages (*p* = 0.017; Figure 2E) and scores 1 and 2 but only in left colon (*p* = 0.002; Figure 2F and Figure S1). As was done previously for clinical features (Table 2), the possible association of nNav1.5 localisation with PFS was also evaluated but no relationship was found (*p* = 0.768; Supplementary Figure S2). Finally, since the presence of several statistically significant associations were found between expression of nNav1.5 and other biomarkers (Table S2), we also investigated the possible impact of biomarker combinations on PFS performing bivariate analyses (Supplementary Figures S3 and S4 and Table S4). Interestingly, although not reaching significance, we observed a clear trend showing that the presence of nNav1.5 had a negative impact on PFS when analysed in combination with all the other biomarkers. This was apparent from the Kaplan–Meier plots where PFS values were generally lower for nNav1.5-expressing cases (blue lines) compared with samples not expressing nNav1.5 (red lines).

## 3. Discussion

In the present paper, we provided evidence for the following: (1) nNav1.5 protein was expressed in colorectal adenocarcinoma samples which, compared with normal mucosa, (i) was at significantly higher levels and (ii) had a more discrete pattern of distribution within cells. (2) Strong immunoreactivity also occurred in adenomas, but this was significantly less discrete than what was observed in adenocarcinomas, i.e., it was more like normal mucosa in being diffuse. (3) nNav1.5 protein was co-expressed with hERG1 and KCa3.1 channels, Glut1 transporter and VEGF-A and EGFR. (4) A statistically significant association was found between nNav1.5 protein expression and TNM stage and the presence of metastases. (5) nNav1.5 expression was inversely correlated with PFS (univariate analysis).

### 3.1. Technical Considerations and Limitations

In the present study, we used the polyclonal antibody NESOpAb to study nNav1.5 protein expression in human colorectal tissues. This antibody was validated extensively at the time of production using recombinant cell lines as well ‘native’ tissues (adult and neonatal heart) [19]. The antibody was successfully re-evaluated more recently as a part of a study on CRCa cells [20]. Here, we made a concerted effort to quantify the immunostaining of the colorectal tissues (normal, adenomas and carcinomas). This was performed using both qualitative and quantitative criteria. Qualitatively, we focused on the pattern of the staining. Quantitatively, both the intensity and the extent of the staining were evaluated. Nevertheless, some limitations remain in these studies. First, although the evaluations were done by two researchers independently, it would have been better to do this ‘blind’. Second, the semi-quantitative staining could have been done more objectively using densitometry. Third, although the quality/pattern of the staining would have been performed better by confocal imaging, IHC involving peroxidase staining is not readily amenable for confocal microscopy and use of immunofluorescence would suffer from the autofluorescence of fixed tissues. It would be desirable to improve the IHC in future studies taking these issues into consideration. Our conclusions would also be strengthened considerably by RNA-seq analyses of gene and transcript expression, including of splice variants. Since our results have significant clinical relevance (Section 3.5), it would also be desirable to simplify the procedures so as to be useable routinely in a hospital diagnostic pathology laboratory. Automated immunostaining techniques are already available, and their quantitation is in development [21,22].

### 3.2. Pathophysiology of VGSC (nNav1.5) Expression in CRCa

House et al. originally demonstrated that the Nav1.5 subtype of VGSC was functionally expressed in CRCa cells [14,23]. Using a pan-Nav1.5 antibody, they also showed that Nav1.5 immunostaining was significantly higher in CRCa tissues compared with normal colon mucosa [14]. This observation has now been extended specifically to nNav1.5 protein expression using a polyclonal antibody (NESOpAb) that had 100-fold greater electrophysiological selectivity for nNav1.5 compared with aNav1.5 [19]. Consistent with the current immunohistochemical observations, Guzel et al. demonstrated in a detailed in vitro study that nNav1.5 mRNA and protein were expressed in several CRCa cell lines and silencing nNav1.5 specifically using several different siRNAs suppressed or eliminated the VGSC-dependent component of invasion [20]. Furthermore, hypoxia increased invasiveness and this was also blocked completely by siRNA targeting nNav1.5 [20]. On the other hand, silencing of aNav1.5 had minimal effect.

In the present study, we demonstrated that nNav1.5 immunoreactivity was significantly higher in adenocarcinomas compared with both matched and unmatched healthy mucosa. An interesting observation was the differing pattern of nNav1.5 expression within individual CRCa biopsy samples. This ranged from diffuse staining throughout the cell to more discrete apical and/or basal expression. Such a difference in the distribution of nNav1.5 was previously reported for breast cancer biopsies where (i) the expression became significantly asymmetrical in cancer biopsies compared to normal tissue and (ii) nNav1.5 expression was exclusively in the plasma membrane for the more clinically aggressive cases [24].

The diffuse staining (low values of DF) observed in some samples, especially adenomas, would imply that for these cases nNav1.5 protein was present mainly in cytoplasm. Such proteins would not normally be functional and become effective once transported to the plasma membrane where channel activity would promote invasiveness via regulation of pericellular pH and proteolysis [25]. Trafficking to the plasma membrane may be controlled by signalling mechanisms such as protein kinase A (PKA) [26,27]. Indeed, PKA (mRNA and protein) levels were found to be significantly increased in CRCa and correlate with invasion depth and shortened survival [28]. In addition, it is also possible under the hypoxic conditions of the adenomas that the channels promote early proliferative activity [29]. Overall, the presence of a significant level of intracellular nNav1.5 protein in adenomas is consistent with their potentially precancerous nature.

nNav1.5 expression was shown to be associated with other CRCa biomarkers (hERG1, KCa3.1, VEGF-A, Glut-1, and EGFR) [30,31,32]. Association with the potassium channels, hERG1 and KCa3.1, is not surprising since ion channels, especially sodium and potassium channels, naturally work in concert. VEGF-A is well known to be involved in angiogenesis and this may be modulated by VGSC activity [33]. EGF has previously been shown to upregulate VGSC expression/activity and to be associated invasiveness in prostate cancer, breast cancer and non-small cell lung cancer [12,34,35]. A similar situation may occur in CRCa where EGF/EGFR represents a major signalling mechanism promoting metastasis [36]. Further work is required to determine the functional consequence(s) of these co-expressions.

### 3.3. nNav1.5 Expression and Survival

nNav1.5 expression was found to correlate significantly with reduced progression-free survival. This is in general agreement with a previous study on Chinese patients [37]. Furthermore, the association of nNav1.5 expression with clinicopathological features (e.g., normal versus cancer tissue, TNM stage and presence of metastases) and progression-free survival are in accordance with what has previously been shown for VGSC expression in other cancers [12,14,24,38,39,40]. Moreover, we provided evidence here that nNav1.5-positive patients have generally a shorter PFS, when analysed in combination with other markers consistent with nNav1.5 expression being the main cause of the PFS lowering. These results complement earlier data from human breast cancer, where nNav1.5 is again dominant, showing that, compared with low-level expression, patients with high levels of (n)Nav1.5 mRNA expression have (i) higher levels of disease recurrence and (i) shorter lifespans [41].

### 3.4. A Model of nNav1.5 Expression in Relation to CRCa Invasiveness

**Figure 3:**
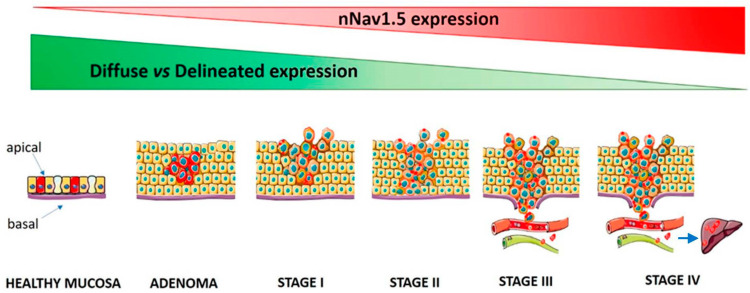
A schematic model illustrating how nNav1.5 expression changes during CRCa progression. Red and green wedges indicate, respectively, increasing expression of functional nNav1.5 protein and decreasing diffuseness/increased delineation of its cellular distribution. Lymphatic vessels are depicted in green while blood vessels are in red. For TNM IV, metastasis to liver is common.

Based on the current findings, we can propose a model for nNav1.5 expression and its role in promoting invasiveness in CRCa (Figure 3).

Thus, in healthy mucosa nNav1.5 is expressed at low levels with a diffuse pattern, localised throughout the cells. In adenomas, the level of expression increases but the pattern of distribution remains the same. For Stage I–III adenocarcinomas, the level of expression is as high as in adenomas, but the protein becomes more focally distributed (apical and/or basal). The same profile is maintained in Stage IV with metastatic cells showing the highest level of expression. These findings are in accordance with the statistically significant association we found between nNav1.5 expression and TNM IV stage and the presence of distant metastases.

### 3.5. Clinical Implications

The available evidence taken together is consistent with nNav1.5 being a viable biomarker of CRCa, especially as it is expressed early in invasiveness [14]. Currently, as noted in the Introduction, the pathology of CRCa is defined by several parameters including TNM stage, *RAS*, *EGFR*, and *BRAF* mutations, and TS, P21, and PTEN protein expression. We can consider adding to this list a composite score of “nNav1.5” expression, e.g., TSxDF (%), as an additional but functional index of metastatic potential. This would be analogous to Ki67, a marker of proliferation, which is also expressed as a percentage and used in evaluating CRCa prognosis [42]. Thus, nNav1.5 expression can be used to better stratify CRCa patients, and this could ensure more effective therapy. Targeted treatment may even be possible using clinically available, ‘repurposed’ VGSC blockers [43,44,45]. As a neonatal splice variant, nNav1.5 has the potential to be ‘cancer specific’ in the adult body [24]. In fact, nNav1.5 is pharmacologically distinguishable from its ‘adult’ counterpart, expressed mainly in cardiomyocytes, so it could be targeted by even more specific blockers and/or an antibody [46]. Thus, nNav1.5 can serve as both a novel drug target and a companion diagnostic tool.

## 4. Materials and Methods

### 4.1. Patients

The cohort analysed in the present study was made of 182 colorectal adenocarcinoma patients who underwent radical surgery with curative intent at three different hospitals (Division of Oncologic Surgery and Robotics, Department of Oncology, Careggi University Hospital, Florence; Department of General Surgery, Campus Bio-Medico University of Rome; and Spedali Civili Hospital, Brescia, Italy). Of 182 consecutively enrolled cases of CRCa, 34 originated from the rectum, 52 from the left colon, 85 from the right colon, and 11 from the transverse colon. The patients encompassed all TNM stages as follows: TNMI (17.00%), TNMII (32.40%), TNMIII (34.60%) and TNMIV (15.90%). The tumour grades were as follows: G1 (5.40%), G2 (92.10%), and G3 (2.40%), with 17 not determined. We also analysed 63 cases of normal mucosa. Of these two groups of tissue, 48 cases were ‘matched’. Finally, 40 cases of adenoma (20 with low- grade dysplasia and 20 with high-grade dysplasia) were studied. All the tissues came with informed written consent and the study was approved by the local ethical committee of Careggi University Hospital (BIO.14.033). All the patients received the appropriate treatment after recurrence or progression, according to the local guidelines. The tissues were fixed in formalin and embedded in paraffin according to established protocols in use at the listed Institutions.

### 4.2. Immunohistochemistry

The procedure was carried out as previously published [13]. In brief, tissue sections were dewaxed for 2 × 15 min in Histoclear (National Diagnostics), rehydrated and endogenous peroxidase activity blocked with 1% H_2_O_2_ (Sigma, Darmstadt, Germany) in methanol (VWR UK) for 10 min. Antigen retrieval (in 0.01 M, pH 6 citric acid buffer for 2 min), preceded blocking of background staining with 10% BSA (Sigma, Darmstadt, Germany) for 1 h. Sections were incubated for 1 h with primary antibody (NESOpAb), specific for nNav1.5 [19], at a dilution of 1:200 from a stock concentration of 0.7 mg/mL. Following washing, biotinylated swine anti-rabbit secondary antibody (DAKO) at a 1:125 dilution was applied for 1 h. All antibody incubations were performed at room temperature. Following completion of the ABC kit protocol DAB staining (Vector Laboratories Inc., Burlingame, CA, USA) and DAB staining (Vector Laboratories Inc.), sections were haematoxylin stained and mounted with DPX. In negative controls, the primary antibody was omitted which eliminated the staining (not shown). Nuclei were counterstained in blue by haematoxylin. Immunohistochemistry for the other biomarkers was performed as previously reported [30,31,32].

### 4.3. Quantitation of the Immunostaining

The immunohistochemical staining of the tissues was quantified by two parameters: (i) Intensity (I) was scored as 0, 1, or 2 (for no, weak and strong staining, respectively) and expressed in arbitrary units (a.u.). (ii) Extent (Ext) of epithelial staining was estimated visually as a percentage of the area stained in randomly chosen fields of view. These assessments were performed by EL and checked independently by either of two other scientists (MG and SPF). There was agreement in more than 90% of cases. Averages were taken in the minority disagreements. We also used a composite parameter for ‘total staining’ (TS) defined as I x Ext (a.u.). The cases of “no staining” were scored as TS = 0. For streamlining the analyses, the TS values were grouped into four classes of “Score” as follows: Score 0 (TS = 0); Score 1 (TS = 1−100); Score 2 (TS = 101−200) and Score 3 (TS = 201−300). In addition, we introduced a parameter, “delineation factor” (DF) to represent the pattern of immunostaining. This was determined as follows: (i) twenty fields of view were chosen randomly for each tissue section; (ii) the staining of each field of view was scored as “discrete” or “diffuse”; and (iii) DF (%) was calculated as [N_D_/20] × 100, where N_D_ is the number of fields of view showing discrete staining. Samples with TS = 0 had to be excluded from this analysis.

### 4.4. Data Analysis

Data were analysed using the statistical software GraphPad Prism 8.0.2 (GraphPad Software Inc., San Diego, CA, USA) and Intercooled Stata 9.1 (StataCorp LP, College Station, TX, USA). Data were analysed by either Mann–Whitney test to compare continuous variables and Chi-square test or Fisher exact test, when appropriate, to compare categorical variables.

Progression free survival (PFS) was defined as the time elapsed between intervention and disease progression or last tumour evaluation and overall survival (OS) was defined as the time elapsed between intervention and death from any cause or last follow-up.

Kaplan–Meier estimated survival curves of PFS and OS were plotted and compared using log-rank test. The impact of clinical and biomolecular parameters on PFS and OS was testes using univariate and multivariate Cox hazard models reporting hazard ratios (HR) and their 95% confidence intervals (95% CI). All statistical tests are two-tailed and a *p*-value < 0.05 was considered statistically significant.

## 5. Conclusions

The data presented here taken together with other available evidence are consistent with nNav1.5 being a viable (early and functional) biomarker of CRCa aggressiveness. Accordingly, nNav1.5 expression can be used to better stratify CRCa patients enabling, in turn, more focussed therapies. Furthermore, nNav1.5 expression being ‘cancer specific’ could be targeted by selective small-molecule drugs (including clinically available, ‘repurposed’ VGSC blockers) and/or an antibody. Thus, nNav1.5 has significant diagnostic and therapeutic potential in the clinical management of CRCa.

## Figures and Tables

**Table 1:** Quantitative data (medians and means) and statistical analyses from the immunohistochemistry. (A) Healthy mucosa, adenomas and adenocarcinomas (Mann Whitney Test). Statistically significant associations (*p* < 0.05) are indicated in bold. (B) Paired healthy colorectal mucosa and adenocarcinoma (Mann Whitney Test). Statistically significant associations (*p* < 0.05) are highlighted in bold. The terminology used are defined in detail in the Materials and methods.

A			*p* Value
**Healthy mucosa, M (*n* = 63)**	Median Extent, E (Mean)	0 (12.10)	M vs. A E: ***p* < 0.0001** SI: ***p* < 0.0001** TS: ***p* < 0.0001** DF: *p* = 0.6462 M vs. ADK E: ***p* < 0.0001** SI: ***p* < 0.0001** TS: ***p* < 0.0001** DF: ***p* < 0.0001** A vs. ADK E: ***p* = 0.0013** SI: ***p* = 0.0006** TS: ***p* < 0.0001** DF: ***p* < 0.0001**
	Median Staining Intensity, SI (Mean)	0 (0.24)	
	Median Total Staining, TS (Mean)	0 (14.68)	
	Median Delineating Factor, DF	0	
**Adenomas, A (*n* = 40)**	Median Extent, E (Mean)	75 (65.25)	
	Median Staining Intensity, SI (Mean)	2 (2.12)	
	Median Total Staining, TS (Mean)	160 (164.5)	
	Median Delineating Factor, DF	0	
**Adenocarcinomas, ADK (*n* = 182)**	Median Extent, E (Mean)	50 (55)	
	Median Staining Intensity, SI (Mean)	1 (2.5)	
	Median Total Staining, TS (Mean)	60 (160)	
	Median Delineating Factor, DF	20	
**B**			
**Paired Healthy mucosa, pM (*n* = 48)**	Median Extent, E (mean)	0 (13.12)	pM vs. pADK E: ***p* < 0.0001** SI: ***p* < 0.0001** TS: ***p* < 0.0001** DF: *p* = 0.1300
	Median Staining Intensity, SI (mean)	0 (0.25)	
	Median Total Staining, TS (mean)	0 (16.46)	
	Median Delineating Factor, DF	0	
**Paired Adenocarcinomas, pADK (*n* = 48)**	Median Extent, E (mean)	25 (33.54)	
	Median Staining Intensity, SI (mean)	1 (0.83)	
	Median Total Staining, TS (mean)	25 (44.17)	
	Median Delineating Factor, DF (mean)	10	

**Table 2:** Association between nNav1.5 expression and clinicopathological parameters. Case numbers are shown with the percentage in brackets. Statistically significant associations (*p* < 0.05) are highlighted in bold (Chi-Square test or Fisher Exact Test).

		nNav1.5 Score 0	nNav1.5 Score 1	nNav1.5 Score 2	nNav1.5 Score 3	*p* Value
**Gender**	Female, 94 (51.60)	29 (55.77)	41 (53.95)	22 (44.90)	2 (40.00)	0.662
	Male, 88 (48.40)	23 (44.23)	35 (46.05)	27 (55.10)	3 (60.00)	
**Localisation**	Right colon, 81 (44.50)	24 (48.08)	31 (42.11)	24 (51.02)	2 (60.00)	0.723
	Transverse, 11 (6.10)	1 (1.92)	8 (10.53)	2 (4.08)	0 (0.00)	
	Left colon, 51 (28.00)	16 (30.77)	19 (26.32)	14 (28.57)	2 (40.00)	
	Rectum, 39 (21.40)	12 (19.23)	18 (21.05	9 (16.33)	0 (0.00)	
**Grading**	G1, 9 (5.40)	5 (10.42)	2 (2.94)	2 (4.55)	0 (0.00)	0.249
	G2, 152 (92.10)	43 (89.58)	65 (95.59)	39 (88.64)	5 (100.00)	
	G3, 4 (2.40)	0 (0.00)	1 (1.47)	3 (6.82)	0 (0.00)	
**TNM stage**	I, 31 (17.00)	10 (19.23)	11 (14.47)	10 (20.41)	0 (0.00)	**0.005**
	II, 59 (32.40)	16 (30.77)	31 (40.79)	12 (24.49)	0 (0.00)	
	III, 63 (34.60)	21 (40.38)	21 (27.63)	21 (42.86)	0 (0.00)	
	IV, 29 (15.90)	5 (9.62)	13 (17.11)	6 (12.24)	5 (100.00)	
**Metastases**	No, 151 (83.00)	46 (88.46)	63 (82.89)	42 (85.71)	0 (0.00)	**<0.001**
	Yes, 31 (17.00)	6 (11.54)	13 (17.11)	7 (14.29)	5 (100.00)	

**Table 3:** Results of PFS and OS univariate analysis for clinical and biomolecular parameters (log-rank test and Mantel Cox test). Statistically significant associations (*p* < 0.05) are highlighted in bold. NE = Not estimable.

		PFS		OS	
		HR (95% CI)	*p*	HR (95% CI)	*p*
**Age**	Continuous variable	0.97 (0.93–0.99)	**0.046**	0.99 (0.96–1.03)	0.687
	<70	1.00	0.174	1.00	0.174
	≥70	0.54 (0.22–1.31)		0.54 (0.22–1.31)	
**Gender**	Female	1.00	0.762	1.00	0.211
	Male	1.14 (0.50–2.59)		1.60 (0.77–3.32)	
**Localisation**	Right colon	1.00	**0.015**	1.00	0.183
	Transverse	0.93 (0.11–7.77)		3.31 (1.14–9.64)	
	Left colon	1.30 (0.40–4.27)		1.44 (0.57–3.59)	
	Rectum	4.22 (1.56–11.43)		1.37 (0.55–3.42)	
**TNM**	I	NE	0.076	NE	0.219
	II	0.29 (0.10–0.85)		0.90 (0.21–3.78)	
	III	0.37 (0.15–0.94)		0.88 (0.24–3.32)	
	IV	1.00		1.94 (0.56–6.70)	
**Metastases**	No	1.00	**0.0008**	1.00	0.702
	Yes	4.06 (1.79–9.34)		0.79 (0.24–2.61)	
**Grading**	G1	NE	0.999	0.78 (0.10–5.75)	0.970
	G2	1.00		1.00	
	G3	0.98 (0.13–7.34)		NE	
**nNav1.5**	Score 0	1.00	**0.031**	1.00	0.716
	Score 1	1.59 (0.45–5.58)		0.79 (0.34–1.82)	
	Score 2	5.96 (1.33–26.65)		0.61 (0.23–1.65)	
	Score 3	4.64 (0.93–23.03)		0.41 (0.05–3.27)	
**hERG1**	Negative	1.00	0.753	1.00	
	Positive	0.88 (0.39–1.99)		0.87 (0.43–1.76)	
**KCa3.1**	Negative	1.00	0.416	1.00	0.950
	Positive	1.72 (0.46–6.36)		0.97 (0.44–2.16)	
**CA IX**	Negative	1.00	0.264	1.00	0.477
	Positive	0.42 (0.09–1.91)		1.32 (0.61–2.85)	
**VEGF-A**	Negative	1.00	0.547	1.00	0.656
	Positive	1.59 (0.35–7.18)		0.82 (0.35–1.93)	
**Glut1**	Negative	1.00	**0.032**	1.00	**0.042**
	Positive	0.11 (0.01–0.82)		0.39 (0.16–0.97)	
**Ki67**	Negative	1.00	0.994	1.00	0.994
	Positive	NE		NE	
**P53**	Negative	1.00	0.412	1.00	0.865
	Positive	1.61 (0.52–4.98)		0.93 (0.43–2.03)	
**Bcl2**	Negative	1.00	0.165	1.00	0.674
	Positive	2.91 (0.64–13.19)		0.73 (0.17–3.11)	
**EGFR**	Negative	1.00	0.899	1.00	0.919
	Positive	0.93 (0.28–3.01)		0.96 (0.43–2.14)	

## Data Availability

The data presented in this study are available on request from the corresponding author.

## Supplementary Materials

The following are available online at https://www.mdpi.com/article/10.3390/cancers13153832/s1, Figure S1: Kaplan-Meier plots of PFS according to nNav1.5 scoring at different localisations in colon, Figure S2: Kaplan-Meier plot of PFS according to the pattern of nNav1.5 expression for all TNM stages, Figure S3: Kaplan-Meier plots of PFS according to the combined expression of nNav1.5, hERG1, nNav1.5 and KCa3.1, Figure S4: Kaplan-Meier plots of PFS according to expression of nNav1.5 in combination with VEGF-A, Glut1 and EGFR, Table S1: Quantitative data and statistical analysis from the immunohistochemistry of nNav1.5 expression in adenomas with low- and high-grade dysplasia, Table S2: Association between nNav1.5 expression and other molecular biomarker expression in CRCa biopsy tissues, Table S3: Associations between nNav1.5 staining pattern and clinicopathological parameters, Table S4: Bivariate analyses of the co-expression of nNav1.5 and other biomarkers (log-rank tests).
