# Supplementary material for: Neonatal Nav1.5 Protein Expression in Human Colorectal Cancer: Immunohistochemical Characterization and Clinical Evaluation
Source: Cancers (Basel). 2021 Jul 30;13(15):3832. doi: 10.3390/cancers13153832 (PMC8345135; doi:10.3390/cancers13153832)
Supplement: Supplementary file 1 [file cancers-13-03832-s001.zip › cancers-1290973-supplementary.pdf]

cancers

Supplementary Material: Neonatal Nav1.5 Protein Expression in Human Colorectal Cancer: Immunohistochemical Characterization and Clinical Evaluation

Elena Lastraioli, Scott P. Fraser, R. Mine Guzel, Jessica Iorio, Lapo Bencini, Emanuela Scarpi, Luca Messerini, Vincenzo Villanacci, Giulia Cerino, Niccolo' Ghezzi, Giuseppe Perrone, Mustafa B. A. Djamgoz and Annarosa Arcangeli

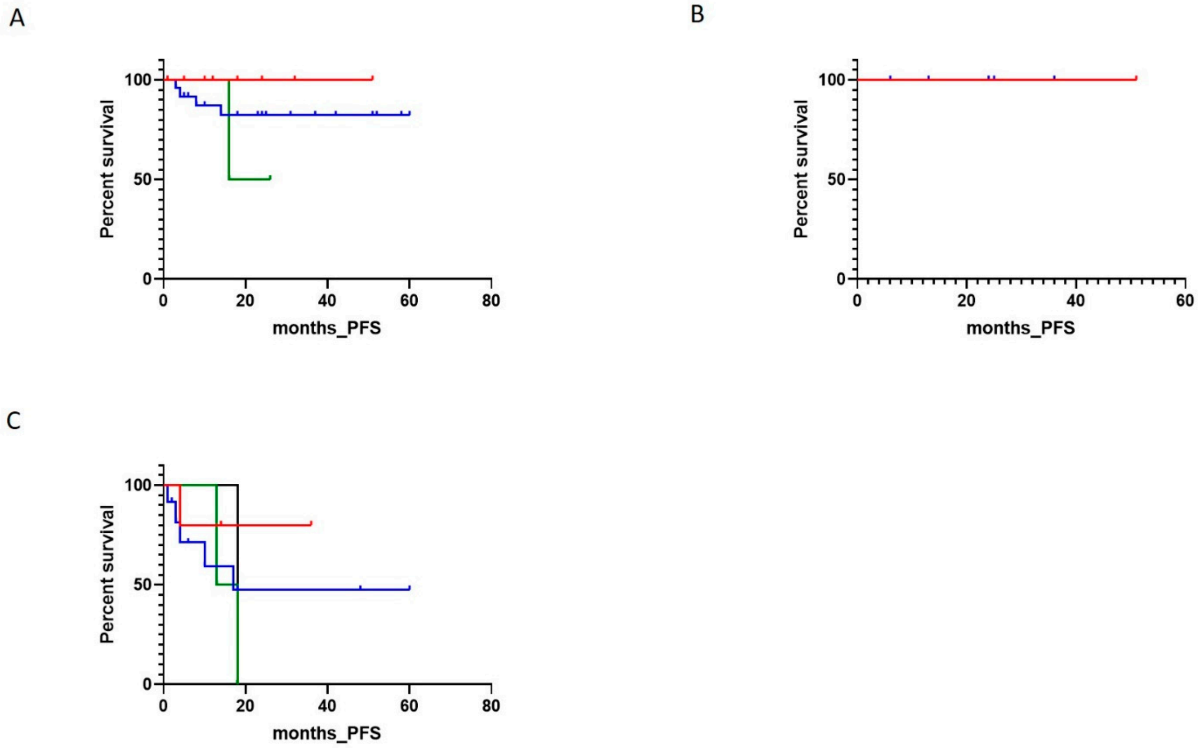

Figure S1. Kaplan-Meier plots of PFS according to nNav1.5 scoring at different localisations in colon. A, right colon; B, transverse colon; and C, rectum. The data for the scores are denoted with different colours: Score 0 (red); Score 1 (blue); Score 2 (green) and Score 3 (black).

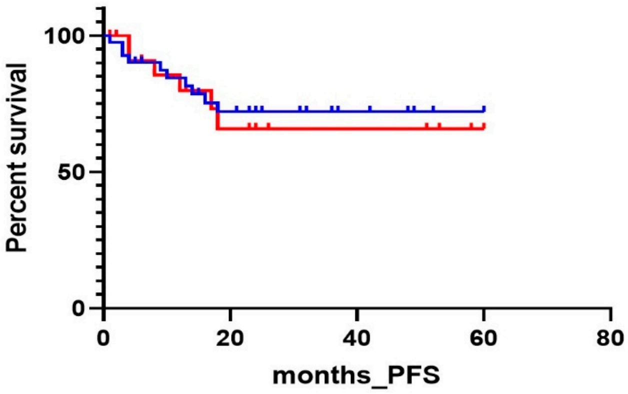

Figure S2. Kaplan-Meier plot of PFS according to the pattern of nNav1.5 expression for all TNM stages. Red lines, delineated expression; blue lines, diffuse expression.

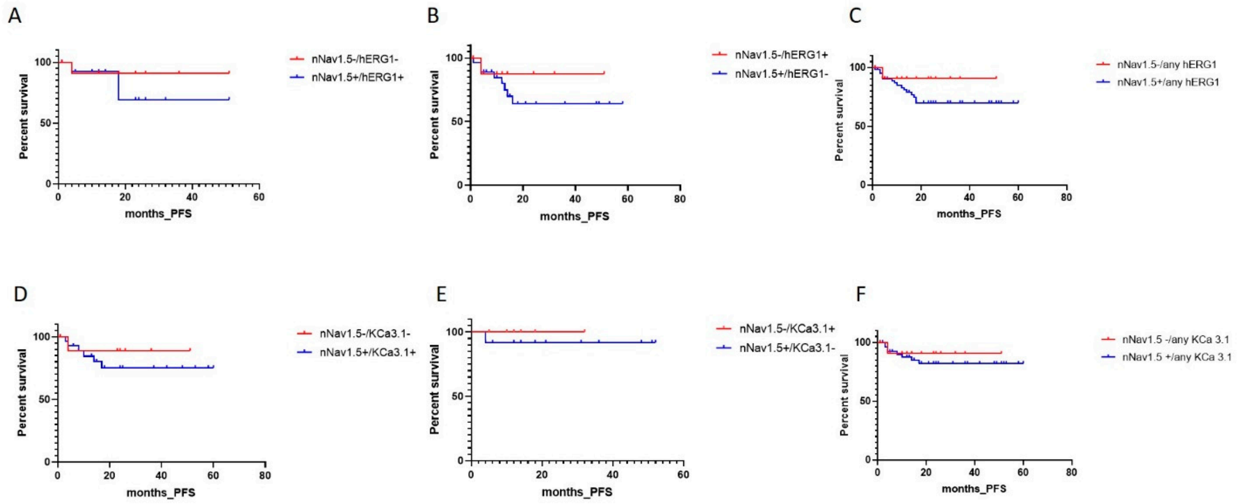

Figure S3. Kaplan-Meier plots of PFS according to the combined expression of nNav1.5 and hERG1 (A-C); and nNav1.5 and KCa3.1 (D-F).

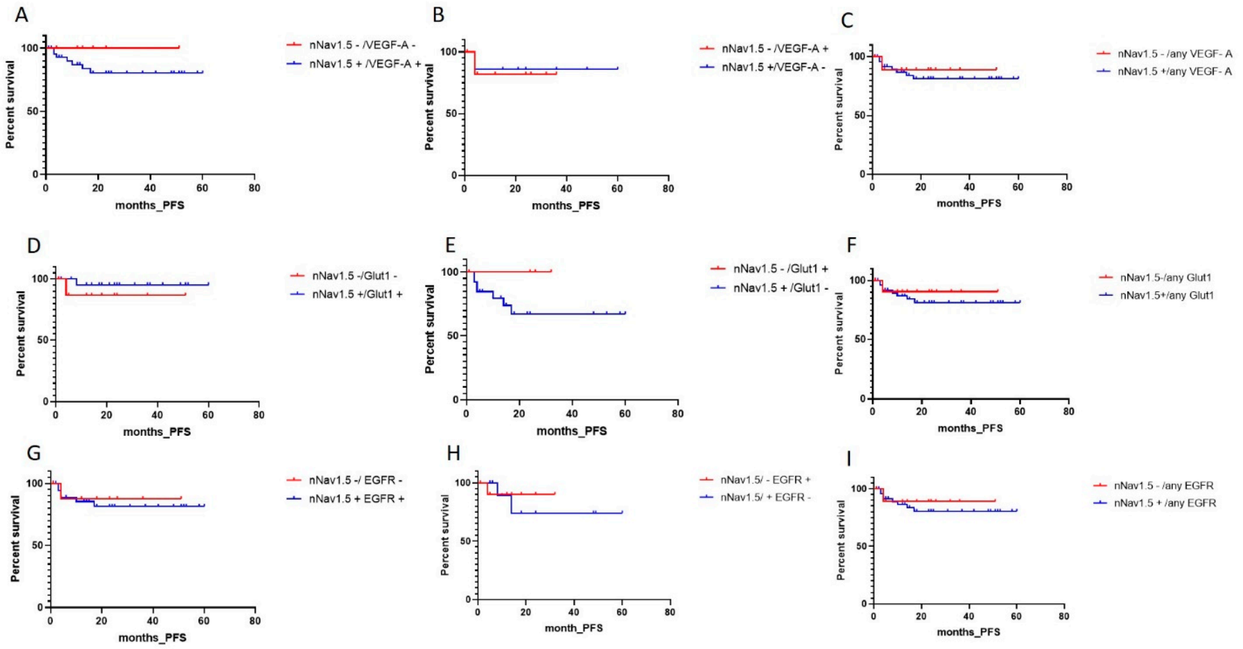

Figure S4. Kaplan-Meier plots of PFS according to expression of nNav1.5 in combination with VEGF-A (A-C); Glut1 (D-F); and EGFR (G-I).

Table S1. Quantitative data and statistical analysis from the immunohistochemistry of nNav1.5 expression in adenomas with low- and high-grade dysplasia (Mann Whitney Test). Data are shown as mean ± SEM. Symbols defined in main text.

Adenoma	Parameter		P value
**Low- grade dysplasia (LGD-A)**	*Extent, E*	61.50±6.78	E: P=0.3294<br>SI: P=0.6099<br>TS: P=0.4299<br>DF: P=1.000
	Staining Intensity, SI	2.05±0.22	
	Median Total Staining, TS	152.50±20.44	
	Median Delineating Factor, DF	0	
**High- grade dysplasia (HGD-A)**	*Median Extent, E*	69.00±6.96	
	Median Staining Intensity, SI	2.20±0.21	
	Median Total Staining, TS	176.50±22.77	
	Median Delineating Factor, DF	0	

Table S2. Association between nNav1.5 expression and other molecular biomarker expression in CRCa biopsy tissues (Chi-Square or Fisher Exact Test). Statistically significant associations (p<0.05) are highlighted in bold.

		nNav1.5 negative	nNav1.5 positive	P value
**hERG1**	*negative*	25 (38.46)	40 (61.54)	**0.046**
	positive	18 (22.78)	61 (77.22)	
**KCa3.1**	*negative*	26 (47.27)	29 (52.73)	**<0.001**
	positive	11 (15.07)	62 (84.93)	
**CA IX**	*negative*	12 (21.05)	45 (78.95)	0.199
	positive	10 (34.48)	19 (65.52)	
**VEGF-A**	*negative*	8 (47.06)	9 (52.94)	**0.032**
	positive	14 (20.29)	55 (79.71)	
**Glut1**	*negative*	18 (33.33)	36 (66.67)	**0.041**
	positive	4 (12.50)	28 (87.50)	
**Ki67**	*negative*	20 (27.40)	53 (72.60)	1.000
	positive	0 (0.00)	2 (100.00)	
**P53**	*negative*	15 (28.85)	37 (71.15)	0.611
	positive	7 (21.88)	25 (78.13)	
**Bcl2**	*negative*	19 (27.14)	51 (72.86)	1.000
	positive	51 (72.86)	5 (83.33)	
**EGFR**	*negative*	10 (43.48)	12 (20.00)	**0.050**
	positive	13 (56.52)	48 (80.00)	

Table S3. Associations between nNav1.5 staining pattern and clinicopathological parameters. Case numbers are shown with the percentage in brackets (Chi-Square or Fisher Exact Test). Statistically significant associations (p<0.05) are highlighted in bold.

		nNav1.5 Negative	nNav1.5 Apical/Basal	nNav1.5 All-over	P value
**Gender**	*Female*	27 (31.03)	24 (27.59)	36 (41.38)	0.836
	Male	25 (31.25)	25 (31.25)	30 (37.50)	
**Localisation**	*Right colon*	33 (40.74)	17 (20.99)	31 (38.27)	**0.027**
	Transverse	0 (0.00)	**6 (60.00)**	4 (40.00)	
	Left colon	12 (24.49)	19 (38.78)	18 (36.73)	
	Rectum	7 (25.93)	7 (25.93)	13 (48.15)	
**Grading**	*G1*	5 (55.56)	2 (22.22)	2 (22.22)	0.325
	G2	43 (31.39)	43 (31.39)	0 (0.00)	
	G3	1 (25.00)	0 (0.00)	3 (75.00)	
**TNM stage**	*I*	11 (37.93)	11 (37.93)	7 (24.14)	0.510
	II	13 (24.53)	16 (30.19)	24 (45.28)	
	III	20 (35.09)	15 (26.32)	22 (38.60)	
	IV	8 (28.57)	7 (25.00)	13 (46.43)	
**Metastases**	*No*	43 (31.39)	41 (29.93)	53 (38.69)	0.905
	Yes	9 (30.00)	8 (26.67)	13 (43.33)	

Table S4. Bivariate analyses of the co-expression of nNav1.5 and other biomarkers (log-rank tests). Only nNav1.5 + Glut1 co-expression reached statistical significance ($p<0.05$), highlighted in bold.

Co-expression	Number of cases ('events')	P value
**nNav1.5- / hERG1-**	1	0.463
**nNav1.5- / hERG1+**	2	
**nNav1.5+ / hERG1-**	10	
**nNav1.5+ / hERG1+**	10	
**nNav1.5- / KCa3.1-**	1	0.765
**nNav1.5- / KCa3.1+**	1	
**nNav1.5+ / KCa3.1-**	2	
**nNav1.5+ / KCa3.1+**	8	
**nNav1.5- / CA IX-**	2	0.654
**nNav1.5- / CA IX +**	1	
**nNav1.5+ / CA IX -**	9	
**nNav1.5+ / CA IX +**	1	
**nNav1.5- / VEGF A-**	1	0.940
**nNav1.5- / VEGF A+**	2	
**nNav1.5+ / VEGF A-**	1	
**nNav1.5+ / VEGF A+**	9	
**nNav1.5- / Glut1-**	3	**0.043**
**nNav1.5- / Glut1+**	0	
**nNav1.5+ / Glut1 -**	9	
**nNav1.5+ / Glut1 +**	1	
